# Unraveling the Role of Microcystin-LR in Ferroptosis and Sepsis Pathogenesis: A Comprehensive Review

**DOI:** 10.3390/biom15070947

**Published:** 2025-06-30

**Authors:** Honglian Ying, Hongli Zhao, Xiaqiu Zhang, Haoyuan Feng, Manjuan Zhou, Zunqiu Wu, Ning Wu

**Affiliations:** Chemistry and Biochemistry Laboratory, Guizhou Medical University, Guiyang 550025, China; 18886355256@163.com (H.Y.); 19985117420@163.com (H.Z.); 19535418172@163.com (X.Z.); 15348647395@163.com (H.F.); 17585162359@163.com (M.Z.)

**Keywords:** sepsis, ferroptosis, Microcystin-LR, oxidative stress, organ injury, immune system, inflammatory factors

## Abstract

Sepsis, a systemic inflammatory response syndrome triggered by infection, is characterized by acute onset, rapid progression, and high mortality. Ferroptosis, a form of programmed cell death induced by iron-dependent lipid peroxidation, is closely associated with the occurrence and development of various diseases. Microcystin-LR (MC-LR) can exacerbate sepsis by causing multi-organ damage via the ferroptosis pathway. Currently, the relationship between MC-LR, ferroptosis, and sepsis remains unclear. Understanding its pathogenesis and identifying potential therapeutic targets may reduce the mortality of sepsis patients and lead to clinical improvement. This article reviews the relationship among MC-LR, ferroptosis, and sepsis, focusing on the mechanism by which MC-LR exposure induces sepsis from the ferroptosis perspective, providing a theoretical basis for the prevention and treatment of MC-LR-exposure-induced sepsis in the population.

## 1. Introduction

Sepsis is a systemic inflammatory response syndrome (SIRS) induced by infection. It is marked by rapid onset and swift disease progression, typically presenting with symptoms such as abnormal body temperature, tachycardia, dyspnea, and irregularities in white blood cell counts. In severe instances, sepsis can progress to septic shock, a life-threatening condition requiring immediate medical intervention [1]. This intricate cascade encompasses several pivotal aspects, notably, the overactivation and dysregulation of the inflammatory response, the disruption and malfunction of immune regulatory mechanisms, the impairment of mitochondrial structure and function, the imbalance between coagulation and anticoagulation systems, aberrant crosstalk within the neuroendocrine-immune network, the activation of endoplasmic reticulum stress responses, and deregulated autophagy processes, among others. These intricately intertwined pathophysiological processes collectively propel the progression of sepsis and ultimately culminate in the dire sequelae of multi-organ dysfunction [2,3]. Despite notable advancements in early diagnosis and treatment strategies for sepsis in recent times, with pertinent research findings being documented [4], the morbidity and mortality rates associated with sepsis persist at high levels [4,5,6], particularly among the elderly, immunocompromised individuals, and patients with chronic diseases, where an upward trend in incidence is evident. Regrettably, the dearth of effective treatments for sepsis underscores the urgent need for collaborative endeavors between the research community and clinical practitioners to achieve a pivotal breakthrough in this field [7].

Ferroptosis, an extraordinary form of cellular demise, is fundamentally distinguished by its iron-mediated instigation of lipid peroxidation [8,9,10,11,12]. This intricate process hinges upon the abnormal accumulation of intracellular iron, leading to a pronounced surge in the levels of toxic lipid peroxides, namely, Reactive Oxygen Species (ROS) [13]. Ferroptosis is intimately linked to the pathophysiological underpinnings of a diverse array of diseases, encompassing but not limited to cancer progression, exacerbated organ damage, and the initiation and progression of neurodegenerative disorders [14,15,16,17,18,19]. Notably, ferroptosis occupies a pivotal position in the pathological landscape of inflammatory responses and sepsis, underscoring the significance of elucidating its regulatory mechanisms for the prevention and therapeutic intervention of associated diseases [20].

Microcystin-LR (MC-LR), a naturally occurring toxin produced by diverse algae genera including *Microcystis*, *Cichlidium*, *Treponema*, and *Cryptococcus*, possesses a distinctive chemical architecture comprising cyclic heptapeptide units featuring unique amino acid side-chains. These toxins exhibit a ubiquitous presence in freshwater ecosystems globally, thereby posing a potential peril to both aquatic ecosystems and human wellbeing [21,22]. In recent years, the scientific research landscape has steadily unveiled an expansive array of detrimental effects attributable to MC-LR. Notably, MC-LR is not merely limited to eliciting severe hepatic injury, but it also manifests the capacity to infiltrate and compromise additional vital organs via the mechanism of ferroptosis, a distinctive cellular demise pathway. This revelation underscores the intricate mechanisms underpinning MC-LR’s participation in the pathological cascade of sepsis, insinuating that it may aggravate sepsis progression by intensifying multi-organ dysfunctionality [23,24].

In light of the above, a thorough exploration of the intricate interplay between MC-LR, ferroptosis, and sepsis holds immense scientific significance and possesses the potential to pave new avenues for the development of clinical diagnostic and therapeutic strategies, alongside enhancing environmental management measures. Nevertheless, the direct nexus between sepsis and MC-LR remains elusive. Consequently, the present study endeavors to delve deeper into the specific mechanistic underpinnings of sepsis triggered by MC-LR exposure, utilizing ferroptosis as a pivotal entry point. By conducting this investigation, we aspire to furnish a robust theoretical foundation and scientific rationale for mitigating sepsis risk associated with MC-LR exposure in populations, while concurrently advancing the quest for effective treatment modalities.

## 2. MC-LR Promotes the Occurrence of Ferroptosis

### 2.1. Mechanism of Ferroptosis

**Figure 1:**
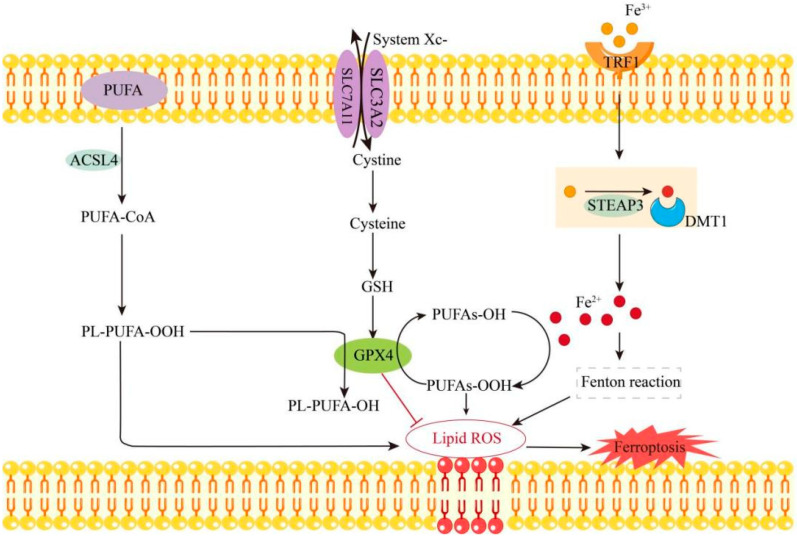
The mechanism of ferroptosis. Fe^3^⁺ in the blood enters cells via TFR1 and is reduced to Fe^2^⁺. ACSL4 assists PUFA integration into phospholipids, enabling their oxidation into toxic lipid peroxides. GPX4 reduces PUFAs-OOH to non-toxic PUFAs-OH, protecting cells from lipid peroxidation damage. However, Fe^2^⁺ can catalyze the conversion of PUFAs-OH to PUFAs-OOH, promoting lipid peroxidation. Meanwhile, Fe^2^⁺ accumulation leads to excessive GSH depletion, reducing GPX4 activity, causing ROS accumulation, catalyzing lipid peroxide buildup, cell membrane rupture, and ultimately, ferroptosis.

Ferroptosis is a form of iron-dependent programmed cell death. Its core mechanisms include abnormal iron metabolism, lipid peroxidation, dysregulation of the antioxidant system, and mitochondrial dysfunction [18,25,26,27]. Transferrin Receptor 1 (TFR1) plays a critical role in ferroptosis by regulating intracellular iron balance through its involvement in iron uptake. Specifically, Fe^3^⁺ in the bloodstream binds to Transferrin (TF) and is transported into the cell. Once recognized by TFR1 on the cell membrane, the iron-TF complex is internalized. Within the endosome, Fe^3^⁺ is reduced to Fe^2^⁺ by the STEAP3 metal reductase. The resulting Fe^2^⁺ then catalyzes the production of ROS via the Fenton reaction, contributing to the ferroptotic process [25]. Acyl-CoA synthetase long-chain family member 4 (ACSL4) is a lipid-metabolizing enzyme that facilitates the incorporation of polyunsaturated fatty acids (PUFAs) into phospholipids. These PUFA-containing phospholipids undergo progressive oxidation, generating toxic lipid peroxides. This process disrupts the structural integrity of biological membranes, ultimately triggering cellular ferroptosis [28]. Reduced glutathione (GSH) is a critical antioxidant and serves as the reducing agent for glutathione peroxidase 4 (GPX4). It neutralizes ROS through direct or enzymatic reactions, during which GSH is oxidized to glutathione disulfide (GSSG). This process reduces ROS levels and protects cells from oxidative stress [29]. GPX4 is a multifunctional protein that can exist in both a free state and as part of a lipid complex. It catalyzes the reduction of toxic lipid hydroperoxides (PUFAs-OOH) into their non-toxic alcohol forms (PUFAs-OH), thereby safeguarding cells from the detrimental effects of lipid peroxidation [30]. However, Fe^2^⁺, transported via divalent metal transporter 1 (DMT1), can also catalyze the conversion of PUFAs-OH to PUFAs-OOH, thereby promoting lipid peroxidation. Additionally, the accumulation of Fe^2^⁺ leads to excessive depletion of reduced GSH. When GSH synthesis is impaired, GPX4 activity declines, resulting in the peroxidation of PUFAs, accumulation of ROS, and increased levels of lipid peroxides. This reduces the cellular antioxidant capacity, causes membrane rupture, and ultimately triggers ferroptosis. In addition, mitochondria undergo significant morphological and functional alterations during ferroptosis, characterized by a reduction in mitochondrial membrane potential and an increase in mitochondrial ROS levels [26]. Ferroptosis is modulated by a variety of signaling pathways. For instance, *p53* promotes ferroptosis by down-regulating the expression of *SLC7A11* [31], whereas *Nrf2* and *HSPB1* suppress ferroptosis by activating antioxidant defense mechanisms [32]. Ferroptosis can be induced by specific compounds, such as Erastin and RSL3, or inhibited by agents like Ferrostatin-1 and Liproxstatin-1 [33,34] (as shown in Figure 1).

### 2.2. Mechanisms Associated with the Promotion of Ferroptosis by MC-LR

MC-LR exerts its toxic effects mainly by inhibiting the activities of protein phosphatase 1 (PP1) and protein phosphatase 2A (PP2A) [35,36]. This inhibition affects a wide range of organs, including the intestine, lungs, heart, brain, and kidneys [37,38,39]. MC-LR is not only potentially carcinogenic and cancer-promoting, but also has immunosuppressive effects and may induce inflammation [30]. Studies have shown that MC-LR can trigger cellular oxidative stress and induce the production of ROS. These ROS are capable of damaging cell membranes, proteins, and DNA, ultimately leading to cell damage and apoptosis [40]. In addition, MC-LR induces down-regulation of *Hamp1* gene expression, leading to impaired mitochondrial membrane function in the organism, and induces the accumulation of iron ions, changes that accompany the development of anemia [41]. These results suggest that MC-LR may exacerbate the induction of ferroptosis.

### 2.3. MC-LR Promotes Ferroptosis

Recent studies have revealed an association between MC-LR and ferroptosis. For example, in an experiment, carp larvae were exposed to different concentrations (0 and 10 μg/L) of MC-LR for 15 days, and as a result, the absence of mitochondrial cristae in the carp intestine was observed, which may indicate impairment of mitochondrial function. This damage was closely related to MC-LR-induced oxidative stress and cell death pathways. Further studies revealed that MC-LR resulted in up-regulation of mRNA for endoplasmic reticulum stress markers and accumulation of iron ions along with GPX4 and GSH activities, thus suggesting that ferroptosis plays an important role in MC-LR-induced intestinal injury [24]. In addition, studies conducted on rats have shown that MC-LR negatively affects renal function, leading to mitochondrial lipid peroxidation through the expression of GPX4 and GSH, which, in turn, induces cellular ferroptosis. The effect of MC-LR has also been studied in the field of gastric injury. It has been shown that MC-LR induces oxidative stress through the antioxidant system, leading to a significant increase in malondialdehyde (MDA) levels, suggesting that ferroptosis may be involved in MC-LR-induced gastric injury [42]. Regarding the neurotoxicity of MC-LR, studies have shown that chronic exposure to MC-LR will activate the *Erk/MAPK* signaling pathway, reduce the GSH/GSSG ratio, and elevate ROS levels, leading to ferroptosis in the brain and resulting in neurotoxic effects [24].

**Figure 2:**
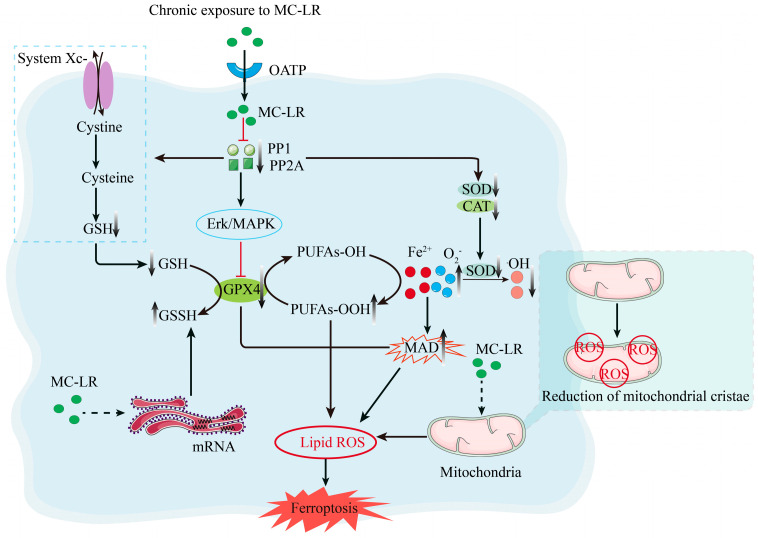
MC-LR promotes the occurrence of ferroptosis. After entering cells via OATP, MC-LR inhibits PP1 and PP2A, suppresses GPX4 activity through the *Erk/MAPK* pathway, and also impairs the antioxidant system. This damages the endoplasmic reticulum and mitochondria, exacerbating lipid peroxidation and iron accumulation, ultimately leading to ferroptosis.

It can be seen that ferroptosis plays a crucial role in the toxic effects of MC-LR and may be the key process of its toxic effects (as shown in Figure 2).

## 3. MC-LR-Associated Pathogenic Pathways in Sepsis Progression

### 3.1. Pathogenesis of Sepsis

Sepsis involves multisystem functional derangements and dysregulated interorgan crosstalk. However, the pathophysiological effects of these systemic interactions and their molecular regulatory networks during sepsis pathogenesis remain mechanistically undefined.

The dysregulated inflammatory response is a central mechanism in the pathogenesis of sepsis. Receptors on inflammatory effector cells are activated by pathogen-associated antigens, triggering the *NF-κB* signaling pathway and up-regulating the expression of pro-inflammatory cytokines, such as Tumor Necrosis Factor-α (TNF-α), Interleukin-6 (IL-6), and Interferon-β (IFN-β) [43]. This cascade culminates in a cytokine storm [44], exacerbating systemic inflammation and contributing to the dysfunction of vital organs [45]. However, in sepsis patients, inflammation triggers a compensatory anti-inflammatory response, leading to immunosuppression and, in severe cases, secondary infections or immune paralysis. Recent studies have shown that most sepsis-related deaths result not from an uncontrolled pro-inflammatory response but from immune paralysis [46]. Pro-inflammatory cytokine elevation further amplifies neutrophil-endothelial adhesion, thereby triggering complement activation and coagulation cascade initiation. This pathophysiological cascade induces thrombocytopenia and disseminated intravascular coagulation (DIC) [47], resulting in microvascular perfusion deficits and multi-organ dysfunction syndrome (MODS). In addition, sepsis concurrently stimulates overproduction of ROS, generating systemic oxidative stress. ROS mediate lipid peroxidation, protein carbonylation, and DNA oxidation, while disrupting endothelial barrier integrity and mitochondrial oxidative phosphorylation. These cumulative effects synergistically drive MODS progression and mortality [48]. In summary, the pathogenesis of sepsis remains incompletely understood. Current research has identified several pathophysiological processes, including dysregulation of the neuroendocrine-immune network, endoplasmic reticulum stress, and autophagy, as critical contributors to the progression of the disease [3].

### 3.2. Molecular Mechanisms of MC-LR-Induced Sepsis

After ingestion, MC-LR is initially absorbed through the intestines, entering the bloodstream by disrupting the epithelial cells of the small intestinal mucosa and the underlying lamina propria. Subsequently, MC-LR is distributed to organs such as the liver, kidneys, and heart, eventually spreading throughout the body [49]. Among these, the liver is the primary target organ for MC-LR-induced toxicity [50]. MC-LR activates innate immunity by polarizing macrophages and neutrophils toward pro-inflammatory phenotypes, thereby triggering systemic inflammation. Upon cellular internalization, MC-LR potently inhibits protein phosphatases PP1/PP2A, dysregulating key intracellular signaling cascades. For instance, MC-LR activates the *PI3K/AKT* pathway to enhance cellular survival and pro-inflammatory cytokine biosynthesis, while concurrent *JAK/STAT* activation exacerbates inflammatory responses [51]. These perturbed pathways converge on *NF-κB* and *MAPK* signaling hubs, amplifying transcriptional activation and secretion of TNF-α, IL-1β, and IL-6 [52,53] The release of inflammatory factors suppresses the *Nrf2* pathway, leading to cellular oxidative stress. For example, studies have demonstrated that the antioxidant α-lipoic acid (α-LA) mitigates MC-LR-induced oxidative stress in mouse liver cells by enhancing *Nrf2/ARE*-mediated antioxidant and detoxifying enzymes. These findings suggest that MC-LR inhibits PP1 and PP2A, activates the *NF-κB* and *MAPK* pathways, and down-regulates *Nrf2* expression, ultimately inducing oxidative stress [54]. This mechanism is highly similar to the pathological causes of sepsis. MC-LR further activates the *NLRP3* inflammasome, inducing caspase-1-mediated proteolytic cleavage of pro-IL-1β and pro-IL-18 into their bioactive forms. This inflammasome-driven cytokine release is a hallmark of sepsis-associated cytokine storms [55].

In addition, MC-LR can trigger apoptosis via the mitochondrial-dependent pathway, leading to the release of mitochondrial DNA (mtDNA) and cytochrome c. This process activates the *cGAS-STING* pathway, further exacerbating the systemic inflammatory response [56]. MC-LR induces mitochondrial dysfunction by causing the collapse of mitochondrial membrane potential and excessive production of ROS [57]. This mitochondrial damage further triggers apoptosis and the release of damage-associated molecular patterns (DAMPs), such as mtDNA and high-mobility group box 1 protein (HMGB1). These DAMPs activate immune cells through *TLR4* and *RAGE* receptors, thereby exacerbating the pathological progression of sepsis [58].

MC-LR can directly impair vascular endothelial cells, increasing vascular permeability and promoting microthrombus formation. Following endothelial cell damage, the release of TF activates the extrinsic coagulation pathway while simultaneously suppressing anticoagulant mechanisms, such as the protein C system. This disruption in coagulation homeostasis leads to DIC [59], a critical contributor to mortality in septic patients [60].

**Figure 3:**
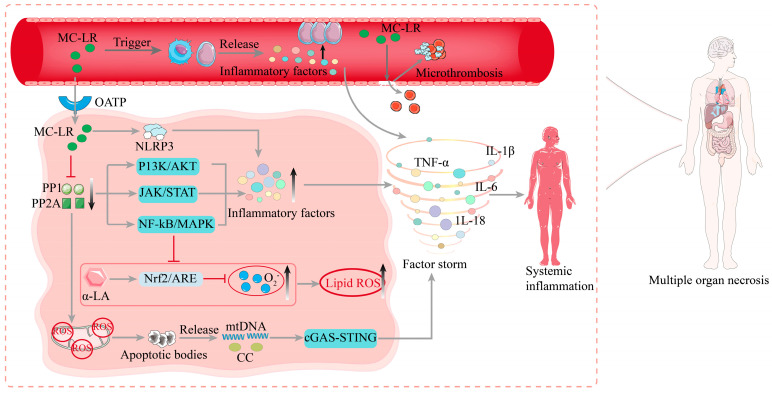
Mechanisms of MC-LR-induced sepsis. Upon entering the bloodstream, MC-LR activates immune cells, triggering the release of inflammatory factors and promoting the adhesion of neutrophils to vascular endothelial cells, directly damaging endothelial cells and increasing vascular permeability and microthrombosis formation. By inhibiting PP1 and PP2A, it activates multiple pathways, induces mitochondrial apoptosis, generates a large number of inflammatory factors, triggers a cytokine storm, and leads to systemic inflammation and multi-organ damage.

In summary, microcystins may trigger sepsis through multiple molecular mechanisms, including immune system activation, dysregulation of cell signaling pathways, cytokine storms, mitochondrial dysfunction, endothelial cell damage, and coagulation abnormalities. These mechanisms are interconnected, forming a complex pathological network that ultimately contributes to MODS and patient mortality (as shown in Figure 3).

## 4. Ferroptosis Contributes to Organ Damage in Sepsis

### 4.1. Sepsis-Induced Liver Injury and Ferroptosis

Acute Liver Failure (ALF) is a severe, multifactorial clinical syndrome characterized by extensive hepatocyte necrosis, uncontrolled systemic inflammation, and insufficient liver regeneration [61,62]. In recent years, increasing evidence has emphasized the significance of ferroptosis in the progression of ALF [63,64]. Studies have shown that ferroptosis in sepsis-induced liver injury in the mouse is associated with the up-regulation of *G protein-coupled receptor 116 (GPR116). GPR116* inhibits the system Xc-/GSH/GPX4 axis, reducing GPX4 activity and GSH synthesis. This exacerbates mitochondrial dysfunction and lipid peroxidation in hepatocytes, ultimately promoting ferroptosis in sepsis-related liver injury [65]. Additionally, research has shown that the expression of *Lipocalin-2 (LCN2)* increases significantly in sepsis-induced liver tissue of mice and lipopolysaccharide (LPS)-treated hepatocytes, mitigating oxidative stress-induced ferroptosis and reducing sepsis-induced liver injury [66], indicating the existence of the ferroptosis pathway in sepsis-induced liver injury.

### 4.2. Sepsis-Induced Kidney Injury and Ferroptosis

Acute Kidney Injury (AKI) is defined as a sudden decline in renal function within 48 h, accounting for 15% of admissions to intensive care units worldwide [67,68]. At the genetic level, *GPX4* knockout mice develop renal failure due to the loss of GPX4’s inhibitory effect on ferroptosis [12]. Studies have reported the presence of ferroptosis in various AKI animal models. Zhang et al. found that sepsis model mice (induced by cecal ligation and puncture, CLP model) exhibited significant AKI indicators 24 h later, with decreased protein expression of ferroptosis marker GPX4, increased protein expression of TF and ferritin heavy polypeptide 1 (FTH1), and elevated serum Fe^2+^ levels. The decrease in GPX4 led to increased mitochondrial oxidative stress (manifested by significantly increased levels of ROS and MDA, as well as significantly decreased levels of GSH and MMP), thereby triggering ferroptosis in glomerular endothelial cells and exacerbating AKI [20]. These results suggest that sepsis may induce ferroptosis by increasing mitochondrial lipid peroxidation and MDA levels while decreasing MMP and GSH, thereby aggravating AKI.

### 4.3. Sepsis-Induced Lung Injury and Ferroptosis

Among all sepsis-induced complications, Acute Lung Injury (ALI) is the earliest and most lethal, with a mortality rate as high as 60% [3,69]. Studies have shown that the imbalance between oxidants and antioxidants plays a crucial role in sepsis-related ALI of murine models [70,71]. Furthermore, ferroptosis is also associated with oxidative stress responses [72]. Additionally, studies have shown that iron and transferrin levels increase significantly in sepsis-induced lung injury [73,74], and MDA levels rise [74]. Research indicates that LPS treatment reduces BEAS-2B cell of human survival, accompanied by down-regulation of ferroptosis markers SLC7A11 and GPX4, and dose-dependent increases in lipid peroxidation products, such as MDA, 4-HNE, and total iron levels [75].

### 4.4. Sepsis-Associated Encephalopathy and Ferroptosis

The Central Nervous System (CNS) is particularly vulnerable to damage mediated by inflammation and oxidative processes, which can lead to Sepsis-Associated Encephalopathy (SAE) [76]. The mechanisms underlying sepsis-associated encephalopathy are well established and encompass oxidative stress, elevated levels of cytokines and pro-inflammatory factors, cerebral circulation disturbances, mitochondrial dysfunction, activation of microglia and astrocytes, and neuronal cell death, etc. [77,78]. In pediatric models of SAE, the accumulation of ROS and down-regulation of GPX4 expression indicate the involvement of the ferroptosis pathway in SAE pathogenesis [79]. Recent studies have further elucidated that the abnormal accumulation of intracellular iron ions represents a potential pathological mechanism in SAE. Specifically, systemic inflammation modulates the expression of cell-surface transporter and receptor genes, primarily characterized by the up-regulation of GPX4 and the down-regulation of ferroportin 1 (Fpn1). This altered expression pattern directly facilitates intracellular iron ion accumulation. Excessive free Fe^2^⁺ can participate in the Fenton reaction, generating ROS that either directly damage cells or induce ferroptosis [80].

### 4.5. Gastrointestinal Injury in Sepsis and Ferroptosis

The gastrointestinal tract, with its extensive mucosal surface and intricate immune mechanisms, is a frontline participant in the systemic response to sepsis [81]. During sepsis, disruption of its barrier function leads to bacterial translocation and leakage of endotoxins into the systemic circulation, further exacerbating inflammatory reactions [82]. Studies have shown that modulating the *Nrf2/GPX4* pathway can effectively intervene in ferroptosis death, intestinal inflammation, and mechanical barrier function in sepsis-induced intestinal injury, indicating the presence of ferroptosis in this context [83]. Additionally, ghrelin has been suggested to protect against sepsis-induced intestinal injury by inhibiting ferroptosis mechanisms: By regulating oxidative stress markers, maintaining mitochondrial structural integrity, up-regulating critical tight junction proteins, and reducing serum levels of intestinal barrier injury markers, such as diamine oxidase (DAO) and fatty acid-binding protein 2 (FABP2), intestinal barrier integrity is enhanced [84].

### 4.6. Septic Cardiomyopathy and Ferroptosis

Septic Cardiomyopathy (SCM), a common and potentially severe complication of sepsis, is characterized by reversible impairment of left ventricular systolic function [85]. This complication significantly worsens patient prognosis, leading to a sharp increase in mortality rates ranging from 70% to 90% [86].

Recent research has uncovered that in SCM mouse models, the expression of a key ferroptosis marker, Prostaglandin-Endoperoxide Synthase 2 (also known as Cyclooxygenase-2, COX-2), is significantly up-regulated in cardiomyocytes. This suggests that sepsis triggers the activation of transferrin on the mitochondrial membrane of cardiomyocytes, which transports iron ions into the mitochondria, disrupting iron metabolism balance within them and ultimately inducing ferroptosis in cardiomyocytes [87].

**Figure 4:**
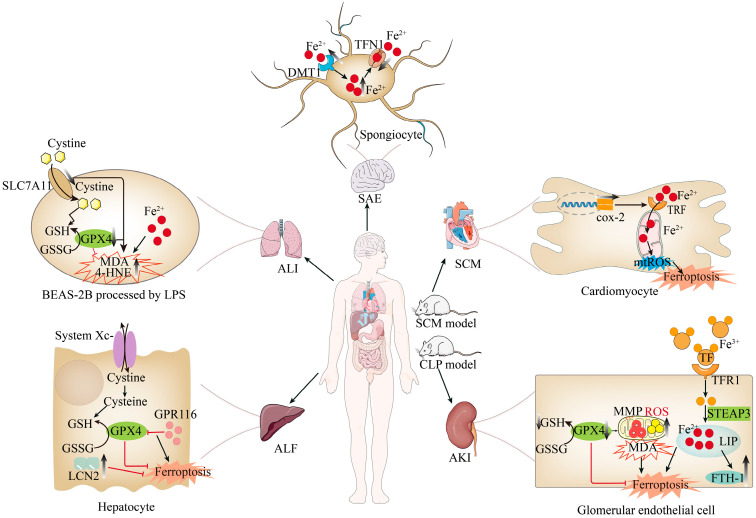
Ferroptosis contributes to multiorgan dysfunction through distinct mechanisms across tissues. The up-regulation of *GPR116* inhibits the expression of the System Xc-/GPX4 pathway, leading to reduced GPX4 activity, which promotes hepatocyte ferroptosis and exacerbates ALF; In LPS-treated lung epithelial cells, the activity of SLC7A11 and GPX4 is reduced, while *TF* activity is increased, leading to enhanced intracellular iron dependency. The accumulation of MDA and 4-HNE promotes ferroptosis in lung epithelial cells, contributing to ALI; increased activity of DMT1 in the brain leads to elevated Fe^2^⁺ influx into glial cells, while the activity of the iron export protein Fpn1 is reduced. This results in the accumulation of Fe^2^⁺ in glial cells, triggering iron overload in glial cells; in renal cells of septic mice, reduced GPX4 activity and decreased GSH production exacerbate mitochondrial lipid peroxidation. Concurrently, increased TF activity enhances the influx of Fe^3^⁺ into cells, which is reduced to Fe^2^⁺ by STEAP3, promoting iron accumulation. The accumulation of lipid peroxidation-related substances (LIP) induces the up-regulation of endogenous FTH-1 transcription. The accumulation of Fe^2^⁺ in renal cells further exacerbates ferroptosis; in the cardiomyocytes of SCM model mice, enhanced gene expression of *COX-2* promotes the transport of iron ions into mitochondria via mitochondrial TF, leading to increased mitochondrial ROS and accelerating the process of myocardial ferroptosis.

In conclusion, ferroptosis plays a pivotal role in sepsis-induced multi-organ injury, and inhibiting ferroptosis can significantly mitigate organ damage, including liver failure, acute kidney injury, acute lung injury, brain injury, intestinal injury, gastric injury, and cardiac injury (as shown in Figure 4).

## 5. MC-LR Induces the Onset of Sepsis Through the Ferroptosis Pathway

Research indicates that MC-LR can cause damage to multiple organs such as the intestine, lungs, heart, brain, and kidneys, and its toxic mechanism is closely related to ferroptosis [9]. Given the strong correlation between ferroptosis and the onset of sepsis, we can reasonably speculate that under the backdrop of multi-organ injury, mechanisms associated with ferroptosis may further induce the pathological progression of sepsis.

After exposure to MC-LR in the intestine, it may disrupt the integrity of the intestinal barrier by inhibiting ghrelin secretion, down-regulating the expression of critical tight junction proteins, and reducing serum levels of intestinal barrier injury markers DAO and FABP2, thereby triggering intestinal damage. Concurrently, MC-LR induces ferroptosis through the *Nrf2/GPX4* pathway by decreasing GPX4 activity and accumulating iron ions, which further exacerbates the damage process and potentially leads to the onset of sepsis [85,86]. Similarly, studies have shown that MC-LR exposure can enter renal cells via organic anion-transporting polypeptides (OATP) [88], potentially triggering ferroptosis by down-regulating the expression of GPX4 and GSH. The reduction in GPX4 leads to increased mitochondrial lipid peroxidation and decreased cytochrome content within mitochondria, which may further amplify oxidative stress and ATP synthesis processes. Excessive mitochondrial lipid peroxidation exacerbates acute kidney injury, potentially contributing to the development of sepsis [20]. Moreover, MC-LR can activate the *Erk/MAPK* signaling pathway, inducing iron accumulation in glial cells and neurons within the brain. This process subsequently triggers cellular inflammation and oxidative stress, leading to the occurrence of sepsis-associated encephalopathy [23].

**Figure 5:**
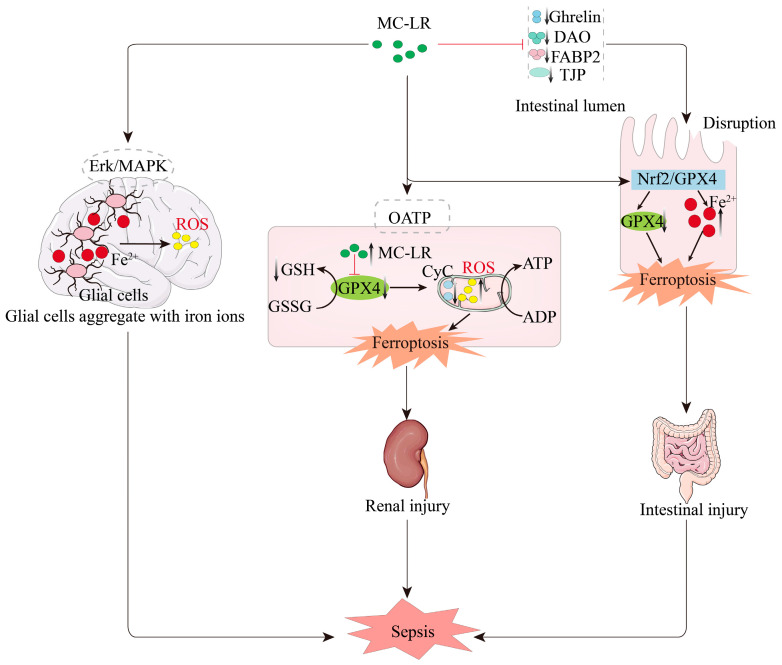
MC-LR induces sepsis through the ferroptosis pathway. MC-LR exposure inhibits the *Nrf2/GPX4* pathway, leading to Fe^2^⁺ accumulation and triggering ferroptosis in intestinal epithelial cells. MC-LR enters renal cells via OATP, suppresses GPX4 expression, reduces GSH production, exacerbates mitochondrial lipid peroxidation, and promotes ferroptosis in renal cells. Simultaneously, MC-LR activates the *Erk/MAPK* pathway, causing Fe^2^⁺ accumulation and increased ROS in brain glial cells, contributing to sepsis-related diseases.

In summary, MC-LR is capable of initiating the ferroptosis process, causing damage to multiple organs, which, in turn, may further induce the onset of sepsis (as shown in Figure 5).

## 6. Conclusions

As a common environmental pollutant, MC-LR poses a significant potential threat to human health. Sepsis is a complex clinical syndrome involving multiple aspects such as unbalanced inflammatory response and immune dysfunction. As a new type of cell death, ferroptosis plays an important role in the pathological process of sepsis.

MC-LR can trigger oxidative stress response in cells by inhibiting the activity of protein phosphatases PP1 and PP2A, leading to organ damage. Especially in important organs such as the liver, kidney, lung, heart, and brain, MC-LR-induced ferroptosis exacerbates the dysfunction of these organs, which may further promote the development of sepsis.

However, the specific relationship between MC-LR and the induction of sepsis through ferroptosis still requires further study. Future research should focus on exploring the mechanism of MC-LR and developing targeted mitigation and treatment strategies. At the same time, improving the efficiency of water management and algal toxin control is also an important aspect of addressing public health challenges.

In summary, MC-LR may exacerbate organ damage in sepsis by inducing ferroptosis. Reducing environmental exposure to MC-LR is an important measure to prevent sepsis. Therefore, in-depth research on the relationship between MC-LR, ferroptosis, and sepsis can help provide scientific evidence for clinical treatment and environmental management. Multidisciplinary collaboration is needed to jointly address this public health challenge.

## Data Availability

No new data were created or analyzed in this study. Data sharing is not applicable to this article.

## Abbreviations

ALF	Acute Liver Failure
AKI	Acute Kidney Injury
ALI	Acute Lung Injury
COX-2	Prostaglandin-Endoperoxide Synthase 2 (Cyclooxygenase-2)
DAO	Diamine Oxidase
DMT1	Divalent Metal Transporter 1
FABP2	Fatty Acid-Binding Protein 2
FTH1	Ferritin Heavy Polypeptide 1
GCLC	Glutamate-Cysteine Ligase Catalytic Subunit
GCLM	Glutamate-Cysteine Ligase Modifier Subunit
GCS	Gamma-Glutamylcysteine Synthetase
GSH	Glutathione
GPX4	Glutathione Peroxidase 4
HAMP1	Hepcidin Antimicrobial Peptide 1
IL-1β	Interleukin-1β
IL-6	Interleukin-6
LCN2	Lipocalin-2
MDA	Malondialdehyde
MC-LR	Microcystin-LR
MDT1	Multidrug Transporter 1
Nrf2	Nuclear Factor Erythroid 2-Related Factor 2
OATP	Organic Anion-Transporting Polypeptides
PP1	Protein Phosphatase 1
PP2A	Protein Phosphatase 2A
PTGS2	Prostaglandin-Endoperoxide Synthase 2
ROS	Reactive Oxygen Species
SAE	Sepsis-Associated Encephalopathy
SCM	Septic Cardiomyopathy
SLC7A11	Solute Carrier Family 7 Member 11
STEAP3	Solute Carrier Family 17 Member 3
TFR1	Transferrin Receptor 1
TNF-α	Tumor Necrosis Factor-α
TJP	Tight Junction Protein
TF	Transferrin
Xc-	System Xc- (Cystine/Glutamate Antiporter)
